# Adaptation and validation of the Carolinas Comfort Scale: a questionnaire-based cross-sectional study

**DOI:** 10.1007/s10029-021-02399-4

**Published:** 2021-03-29

**Authors:** A. Parseliunas, S. Paskauskas, V. Simatoniene, J. Vaitekunas, D. Venskutonis

**Affiliations:** 1 Department of General Surgery, Lithuanian University of Health Sciences, A. Mickevičiaus g. 9, LT-44307 Kaunas, Lithuania; 2 Department of Obstetrics and Gynecology, Lithuanian University of Health Sciences, A. Mickevičiaus g. 9, Kaunas, LT-44307 Lithuania; 3 Department of Physics, Mathematics and Biophysics, Lithuanian University of Health Sciences, A. Mickevičiaus g. 9, Kaunas, LT-44307 Lithuania

**Keywords:** Abdominal hernia, Inguinal hernia, Quality of life, Carolinas Comfort Scale

## Abstract

**Purpose:**

Quality of life (QoL) is an important outcome following surgery. The Carolinas Comfort scale (CCS) is a specific questionnaire used to evaluate QoL in patients who underwent abdominal hernia repair with mesh. The aim of this study was to create a Lithuanian version of the CCS.

**Methods:**

A questionnaire-based cross-sectional study was conducted. A Lithuanian version of the CCS was created by translating the original questionnaire in accordance with the guidelines. The Lithuanian questionnaire was provided to hernia patients at 1 week and at 1 month postoperatively. The main validation characteristics of the Lithuanian CCS were assessed and compared to the original version.

**Results:**

The complete response rate of patients was close to 90%. Internal consistency was excellent, with a Cronbach’s *α* of 0.953. Correlation coefficients ranged from 0.361 to 0.703 in the test–retest analysis. In the construct validity analysis, the strongest correlations were observed in the domains of physical functioning and bodily pain (− 0.655 and − 0.584, respectively) and the weakest correlations in role-emotional and mental health (− 0.268 and − 0.230, respectively). The mean scores of all CCS domains and the total score for satisfied patients were significantly lower (*p* < 0.001) than those of dissatisfied patients. The principal component analysis identified 3 components, with the first accounting for 56% of the variance.

**Conclusions:**

The Lithuanian version of CCS maintains the original validity and is a reliable and valid tool for assessing specific QoL factors after the repair of inguinal hernia with mesh. We recommend using this CCS version in personal, local, and international contexts.

**Supplementary Information:**

The online version contains supplementary material available at 10.1007/s10029-021-02399-4.

## Introduction

Surgical repair of an abdominal hernia is the most common procedure in general surgery [1, 2]. The use of the mesh repair technique has reduced recurrence rates to acceptable and consistent levels [1, 3]. The implant remains in the body for the remainder of the patient’s life and can lead to mesh-associated symptoms, such as foreign body sensation, chronic pain, and long-term physical and mental impairment [1, 4]. Quality of life (QoL) is the most important patient-centered outcome following hernia surgery. QoL questionnaires allow comparison of different surgical techniques and patient outcomes between surgery departments throughout the world.

SF-36 is considered the global gold standard for assessing general QoL in patients with different clinical conditions, including those who have undergone hernia repair with or without mesh [1, 3, 4]. However, it has been shown that SF-36 has limited sensitivity for patients undergoing hernia surgery and is not an adequate measure of QoL in patients who suffer from a chronic condition [3, 4]. Disease-specific questionnaires are superior for detecting changes caused by surgical treatments [3, 5, 6].

The Carolinas Comfort Scale (CCS) is a well-studied and validated disease-specific questionnaire for patients who underwent mesh hernia repair [1, 3, 4, 7]. It was shown to be a superior tool for QoL assessment in these patients compared to SF-36 [1, 3, 4, 6]. CCS is currently used worldwide in more than 48 countries, has been translated into 25 languages, and can be completed online or by mail [1, 3, 7]. Additionally, CCS provides the opportunity to investigate changes over time in a patient’s QoL during the rehabilitation period. Therefore, CCS can be used preoperatively and postoperatively and at various time intervals [1–4, 8].

The main purpose of this study was to adapt and validate a Lithuanian version of the CCS and to compare it with the Lithuanian version of SF-36. A Lithuanian version of the CCS is needed to allow a comparison of the results of hernia surgery in regional and international contexts, as well as to allow participation in hernia clinical trials.

## Materials and methods

### Translation and cross-cultural adaptation

The development of a Lithuanian version of the CCS consisted of three main steps as recommended by Sousa and Rojjanasrirat [9]. Firstly, two independent translators who were native Lithuanian speakers and bilingual translated the original CCS into two Lithuanian versions of CCS. One translator was a physician well versed in health terminology, while the other was a non-medical professional translator familiar with cultural and linguistic nuances. A single version of the Lithuanian CCS was created by a committee of 3 physicians, one medical student and one member from the Linguistics department of the Lithuanian University of Health Sciences.

Next, a reverse translation from Lithuanian back to English was performed by two interpreters. One was a native English speaker also fluent in Lithuanian, while the other was a professional translator and native Lithuanian speaker. The committee compared the back translation to the original CCS version. No major differences were found regarding the format, wording, grammatical structure of sentences, or similarity in meaning. The primary version of the Lithuanian CCS was thus created.

A pilot study with the primary version of Lithuanian CCS was performed in a group of 15 patients who underwent hernia repair using mesh, as previously recommended [10, 11]. An additional question (‘‘Do you understand the question?’’) was added to each CCS item. A number of participants (6/15, 40%) indicated the 8th question was confusing since the term “exercising” in Lithuanian has several possible meanings. Therefore, this question was re-discussed by the committee. In order to make the questionnaire more linguistically accurate, the word “exercising” in the 8th question was changed to a more suitable synonym (“atlikdami pratimus” was changed to “mankštindamiesi”). An additional pilot study was conducted with 10 participants. All questions were understandable for all participants and no corrections were needed. Consequently, the committee, with the consent of the authors for the original questionnaire, approved the final Lithuanian CCS version (Appendix 1 in ESM).

## Clinical validation

### Participants and data collection procedure

The study was conducted from August 2018 to December 2019 in the Department of General Surgery at the Lithuanian University of Health Sciences. This was a questionnaire-based cross-sectional survey in a group of patients who underwent various abdominal wall hernia mesh repairs. The study was approved by the Kaunas Regional Biomedical Research Ethics Committee (protocol number BE-2-44; 2018-06-05).

The inclusion criteria were: age 18–75 years; elective laparoscopic or open surgical repair of inguinal, umbilical, primary ventral or incisional hernia using a mesh; American Society of Anesthesiologists physical status I–III; no cognitive, language, hearing or visual disorders; no movement disorders; native Lithuanian speakers. The exclusion criteria were: refusal to participate; cognitive or physical condition that limited their capacity to answer the questionnaire.

**Fig. 1:**
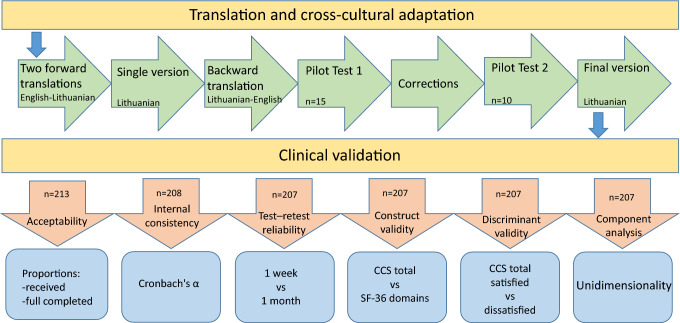
Schematic diagram of the study design

Eligible patients were invited by the investigator to participate in the study and signed an informed consent form. Demographic and clinical data for each participant was collected pre- and peri-operatively using a predesigned questionnaire. Before each participant was discharged, they were briefly instructed on how to complete the SF-36 and CCS questionnaires. The two questionnaires were mailed to participants 1 week and 1 month postoperatively, together with stamped, self-addressed envelopes. Participants completed the questionnaires and mailed them back to investigators. This method has been validated against in-person responses or replies obtained by telephone and has been successfully used in previous trials [1, 3, 12]. If the returned questionnaire was incomplete and there were one or two missing values, it was completed using a scoring algorithm that estimates missing values. If more than two values were missing, participants were called by phone and the missing values were completed during the interview. At the 1-month time point, participants were required to answer an additional question on whether they were satisfied with the QoL as it pertains to hernia repair. A schematic diagram of the study design is presented in Fig. 1.

### Questionnaires

The CCS questionnaire has 23 items for the assessment of health-related QoL after hernia repair with mesh. The score for each item is recorded on a Likert-type scale. CCS evaluates QoL during the course of 8 activities: lying down, bending over, sitting up, activities of daily living, coughing or deep breathing, walking, climbing stairs, and exercise. The total score is based on a scale of 0–115. The higher the score the lower is the health-related QoL.

The SF-36 short form health survey is a well-known, reliable and valid general QoL measuring instrument that is already used in the Lithuanian population with different clinical conditions [13, 14]. SF-36 includes 8 multiple-item subscales that evaluate the physical function, social functioning, role limitations due to physical problems, role limitations caused by emotional problems, mental health, vitality, pain, and general health perception. The total score on each SF-36 subscale ranges between 0 and 100, with a higher score indicating better QoL.

### Statistical analysis

Statistical analysis was performed using SPSS version 22 software (SPSS, Inc., Chicago, IL, USA). A significance level of *p* < 0.05 was used. Baseline characteristics and demographic data were analyzed descriptively. Continuous data are presented as mean and standard deviation, while categorical data are presented as a percentage. Participants were divided into two groups according to the location of their hernia: the “inguinal hernia” group and the “other abdominal hernia” group, which included participants with umbilical, ventral, and incisional hernias. The item characteristics, internal consistency, and test–retest reliability were assessed separately for both groups, as well as in all participants grouped together.

#### Acceptability of the questionnaire

Acceptability was measured as the proportion of returned and fully completed questionnaires. Returned questionnaires having at least one missing item were considered incomplete.

#### Scale and item characteristics

Mean, standard deviation, and the internal consistency of scale and each item, item-total correlations (correlation between an item and the scale that is composed of other items) were calculated to determine item characteristics.

#### Internal consistency

Internal consistency was assessed using the Cronbach α coefficient, which summarizes the internal correlations of all items in a scale [15]. The higher the coefficient (range 0–1), the more consistent is the scale and the greater the likelihood that it is tapping an underlying single variable in the questionnaire. A value of ≥ 0.7 indicates high reliability; 0.5 to < 0.7, moderate reliability; > 0.2 to < 0.5, fair reliability; and ≤ 0.2, low reliability.

#### Reliability

Test–retest reliability was estimated by the interclass correlation coefficient *r* (ICC) (Two-way Random Effect Model Absolute Agreement Definition) of two assessments completed 3 weeks apart from each other (1 week to 1 month postoperatively). Reproducibility was considered to be “excellent” (*r* > 0.75), “good” (0.75 < *r* < 0.40), or “poor” (*r* < 0.40) [16].

#### Construct validity

Evidence for construct validity was obtained from a priori hypothesized patterns of associations with other validated instruments used to measure relatively similar constructs (for positive correlations) [17]. The validity of the CCS was determined by comparing CCS scores with the different domain scores from SF-36 at different time points using Spearman’s rank correlation coefficient (*r*), as done previously for validation of the Dutch CCS version [1]. Strong correlation was considered as values > 0.50, moderate correlation as values between 0.35 and 0.50, and weak correlation as values < 0.35 [18].

#### Discriminant validity

Discriminant validity explores the ability of CCS to discriminate between patients who are satisfied with the results of their hernia repair from those who are dissatisfied. Discriminant validity was assessed by comparing the CCS scores of different domains for patients who were satisfied or dissatisfied at one month after surgery. Mean values were compared by the Mann–Whitney *U* test.

#### Principal component analysis

Principal component analysis was performed to determine whether CCS is uni-dimensional and whether questions in the CCS could be removed.

## Results

A total of 213 patients participated in this study, of which 168 participants underwent inguinal hernia surgery and 45 underwent other abdominal hernia surgery using mesh (22 incisional, 18 umbilical, 5 other ventral hernias). Basic characteristics of the participants are shown in Table 1 and response rates and acceptability results are shown in Table 2. The completion rate was high and did not correlate with the hernia type.

**Table 1:** Basic characteristics of participants undergoing surgery for hernia

Characteristics	All hernias *n* = 213	Inguinal hernia *n* = 168 (78.9%)	Other abdominal hernias *n* = 45 (21.2%)
Age (Mean ± SD)	56.56 ± 13.01	55.73 ± 13.43	59.64 ± 10.90
Sex *n* (%)			
Male	183 (85.9)	158 (94.0)	25 (55.6)
Female	30 (14.1)	10 (6.0)	20 (44.4)
BMI (Mean ± SD)	27.53 ± 5.03	25.78 ± 3.09	33.97 ± 5.56
ASA Physical status n (%)			
I	79 (37.1)	72 (42.9)	7 (15.6)
II	103 (48.4)	80 (47.6)	23 (51.1)
III	31 (14.6)	16 (9.5)	15 (33.3)
Surgery type *n* (%)			
Open	122 (57.3)	77 (45.8)	45 (100)
Laparoscopic	91 (42.7)	91 (54.2)	0 (0.0)
Surgery duration (min) (Mean ± SD)	82.73 ± 26.56	82.27 ± 23.35	84.43 ± 36.33
Hernia character *n* (%)			
Primary	206 (96.7)	164 (97.6)	42 (93.3)
Recurrent	7 (3.3)	4 (2.4)	3 (6.7)
Hernia size EHS* *n* (%)			
1		20 (12.4)	–
2		88 (54.7)	–
3		53 (32.9)	–

^a^Hernia size according to EHS classification [19]

**Table 2:** Participant response and completion rates, *n* (%)

	All hernias (*n* = 213)		Inguinal hernia (*n* = 168)		Other abdominal hernias (*n* = 45)	
Timepoint	Received	Fully completed	Received	Fully completed	Received	Fully completed
1 week	208 (97.7%)	184 (88.5%)	164 (97.6%)	150 (91.5%)	44 (97.8%)	36 (81.8%)
1 month	207 (97.2%)	175 (84.5%)	163 (97.0%)	138 (84.7%)	44 (97.8%)	37 (84.1%)

The CCS showed excellent internal consistency, with a Cronbach’s α coefficient of 0.953 for the overall group, 0.954 for inguinal hernia and 0.951 for other abdominal hernias (Table 4). When a single variable was deleted, the Cronbach’s *α* coefficient ranged from 0.949 to 0.957 in the overall group, 0.924–0.929 in the inguinal hernia group, and 0.944–0.949 for other abdominal hernias (Table 3).

**Table 3:** Reliability Scale (Cronbach's *α* if item was deleted)

Domain	Content	All hernias	Inguinal hernia	Other abdominal hernias
Laying down	Sensation of mesh	0.952	0.953	0.949
	Pain	0.952	0.953	0.949
Bending over	Sensation of mesh	0.950	0.951	0.948
	Pain	0.950	0.951	0.947
	Movement limitations	0.949	0.950	0.947
Sitting up	Sensation of mesh	0.951	0.951	0.949
	Pain	0.949	0.950	0.947
	Movement limitations	0.950	0.951	0.948
Activities of daily living	Sensation of mesh	0.951	0.952	0.948
	Pain	0.949	0.950	0.947
	Movement limitations	0.950	0.950	0.949
Coughing or deep breathing	Sensation of mesh	0.950	0.951	0.949
	Pain	0.950	0.950	0.949
	Movement limitations	0.950	0.950	0.948
Walking	Sensation of mesh	0.950	0.951	0.948
	Pain	0.950	0.951	0.947
	Movement limitations	0.950	0.951	0.948
Walking up the stairs	Sensation of mesh	0.950	0.951	0.949
	Pain	0.950	0.951	0.948
	Movement limitations	0.950	0.951	0.947
Exercising	Sensation of mesh	0.957	0.957	0.956
	Pain	0.954	0.955	0.952
	Movement limitations	0.954	0.955	0.952

Table 4 shows the results for the mean, standard deviation, and internal consistency of the CCS domains, and item-total correlation. The Cronbach’s α coefficient ranged from 0.745 to 0.958 for all items (Table 4).

**Table 4:** Mean, standard deviation (SD), internal consistency and item-total correlation of CCS domains

Domain	All hernias			Inguinal hernia			Other abdominal hernias		
	Mean ± SD	Mean of inter-item correl	Cronbach ‘s α	Mean ± SD	Mean of inter-item correl	Cronbach ‘s *α*	Mean ± SD	Mean of inter-item correl	Cronbach ‘s *α*
Laying down	0.91 ± 1.24	0.608	0.751	0.89 ± 1.20	0.603	0.745	1.00 ± 1.36	0.626	0.768
Bending over	2.86 ± 2.69	0.634	0.838	2.87 ± 2.36	0.626	0.835	2.80 ± 2.55	0.665	0.850
Sitting up	2.06 ± 2.31	0.671	0.860	2.09 ± 2.31	0.702	0.876	1.93 ± 2.33	0.566	0.801
Activities of daily living	2.94 ± 2.39	0.599	0.821	2.85 ± 2.35	0.620	0.833	3.16 ± 2.51	0.535	0.784
Coughing or deep breathing	2.91 ± 2.86	0.697	0.874	2.89 ± 2.84	0.722	0.887	2.98 ± 2.97	0.620	0.834
Walking	2.21 ± 2.26	0.621	0.833	2.27 ± 2.20	0.603	0.821	1.98 ± 2.48	0.686	0.870
Walking up the stairs	2.28 ± 2.24	0.674	0.863	2.33 ± 2.23	0.675	0.863	2.09 ± 2.31	0.674	0.863
Exercising	4.43 ± 4.67	0.881	0.956	4.29 ± 4.50	0.880	0.956	4.93 ± 5.29	0.886	0.958
Total	20.59 ± 16.47	0.527	0.953	20.52 ± 16.18	0.527	0.954	20.86 ± 17.71	0.533	0.951

In the reliability (test–retest) analysis, correlation coefficients between the two separate administrations for each question in the CCS ranged from 0.361 to 0.703, with all correlations being pronounced (Table 5).

**Table 5:** Test–retest reliability

Domain	Content	All hernias		Inguinal hernia		Other abdominal hernias	
		Correlation coefficient	95% Confidence interval	Correlation coefficient	95% Confidence interval	Correlation coefficient	95% Confidence interval
Laying down	Sensation of mesh	0.440**	0.297–0.574	0.461**	0.310–0.614	0.365*	0.054–0.658
	Pain	0.387**	0.234–0.515	0.393**	0.241–0.526	0.361*	0.089–0.628
Bending over	Sensation of mesh	0.543**	0.423–0.655	0.506**	0.372–0.636	0.702**	0.470–0.890
	Pain	0.389**	0.271–0.509	0.363**	0.221–0.492	0.479*	0.129–0.726
	Movement limitations	0.482**	0.379–0.582	0.426**	0.299–0.546	0.703**	0.503–0.824
Sitting up	Sensation of mesh	0.483**	0.344–0.609	0.444**	0.293–0.583	0.622**	0.357–0.861
	Pain	0.397**	0.281–0.518	0.384**	0.248–0.506	0.466*	0.149–0.718
	Movement limitations	0.388**	0.273–0.496	0.392**	0.259–0.506	0.415*	0.109–0.668
Activities of daily living	Sensation of mesh	0.520**	0.391–0.622	0.501**	0.366–0.627	0.622**	0.374–0.822
	Pain	0.455**	0.343–0.559	0.429**	0.289–0.555	0.552**	0.305–0.746
	Movement limitations	0.500**	0.400–0.597	0.479**	0.365–0.586	0.594**	0.314–0.795
Coughing or deep breathing	Sensation of mesh	0.590**	0.472–0.700	0.568**	0.438–0.690	0.690**	0.464–0.858
	Pain	0.409**	0.293–0.523	0.404**	0.266–0.534	0.475*	0.179–0.689
	Movement limitations	0.528**	0.424–0.618	0.533**	0.415–0.640	0.513**	0.239–0.721
Walking	Sensation of mesh	0.494**	0.368–0.623	0.504**	0.336–0.639	0.476*	0.156–0.745
	Pain	0.456**	0.326–0.572	0.499**	0.366–0.608	0.353*	0.005–0.634
	Movement limitations	0.421**	0.294–0.539	0.423**	0.285–0.546	0.478*	0.153–0.729
Walking up the stairs	Sensation of mesh	0.603**	0.482–0.708	0.585**	0.438–0.700	0.690**	0.427–0.893
	Pain	0.442**	0.308–0.559	0.483**	0.355–0.589	0.337*	0.041–0.600
	Movement limitations	0.511**	0.410–0.609	0.511**	0.403–0.615	0.566**	0.353–0.757
Exercising	Sensation of mesh	0.491**	0.363–0.608	0.501**	0.361–0.635	0.483*	0.171–0.754
	Pain	0.416**	0.293–0.529	0.380**	0.230–0.516	0.524**	0.263–0.744
	Movement limitations	0.523**	0.403–0.647	0.502**	0.368–0.620	0.633**	0.357–0.842

*Correlation is significant, *p* < 0.05

**Correlation is significant, *p* < 0.01

In the construct validity analysis, all correlations between different domains of SF-36 and the total CCS score were significant. However, the strongest correlations were found in the domains for physical functioning and bodily pain, and hence the physical component summary score. Similar correlations were observed when the data were analyzed according to hernia type. Correlations at 1 week postoperative were also similar to those at 1 month postoperative (Table 6).

**Table 6:** Construct validity (correlation CCS total score vs. SF-36 domain scores)

SF-36 domains	1 Week			1 Month		
	Correlation coefficient	95% confidence interval		Correlation coefficient	95% confidence interval	
Physical functioning	− 0.563*	− 0.663	− 0.450	− 0.655*	− 0.747	− 0.549
Role-physical	− 0.387*	− 0.501	− 0.261	− 0.505*	− 0.603	− 0.390
Bodily pain	− 0.620*	− 0.699	− 0.530	− 0.584*	− 0.677	− 0.470
General health	− 0.290*	− 0.411	− 0.153	− 0.165**	− 0.298	− 0.031
Vitality	− 0.360*	− 0.487	− 0.232	− 0.336*	− 0.446	− 0.206
Social function	− 0.493*	− 0.602	− 0.379	− 0.454*	− 0.567	− 0.334
Role-emotional	− 0.268*	− 0.393	− 0.131	− 0.408*	− 0.525	− 0.292
Mental health	− 0.230*	− 0.356	− 0.101	− 0.244*	− 0.372	− 0.108
PCS	− 0.628*	− 0.710	− 0.535	− 0.594*	− 0.684	− 0.487
MCS	− 0.430*	− 0.536	− 0.310	− 0.442*	− 0.548	− 0.324

*MCS* mental component summary score, *PCS* physical component summary score

*Correlation is significant, *p* < 0.001

**Correlation is significant, *p* = 0.017

Discriminant validity analysis revealed that 17/207 (8.2%) participants were not satisfied with their hernia-related QoL. For satisfied patients, the mean scores of all CCS domains as well as the total score were significantly (*p* < 0.001) lower than those for dissatisfied patients (Table 7). Significant differences were also found in all SF-36 domains at both time points, except for mental health and general health at 1 month (Table 8).

**Table 7:** CCS scores (mean ± SD) for satisfied and dissatisfied participants

Domain	1 Week			1 Month		
	Satisfied	Dissatisfied	*p*	Satisfied	Dissatisfied	*p*
Laying down	0.81 ± 1.17	2.00 ± 1.46	< 0.001	0.41 ± 0.73	1.94 ± 1.73	< 0.001
Bending over	2.56 ± 2.08	5.94 ± 3.21	< 0.001	1.09 ± 1.30	4.83 ± 3.11	< 0.001
Sitting up	1.79 ± 1.94	4.89 ± 3.72	< 0.001	0.66 ± 1.00	3.72 ± 3.16	< 0.001
Activities of daily living	2.70 ± 2.15	5.50 ± 3.29	< 0.001	1.10 ± 1.33	5.00 ± 3.20	< 0.001
Coughing or deep breathing	1.94 ± 1.97	5.06 ± 3.19	< 0.001	0.85 ± 1.31	4.28 ± 2.95	< 0.001
Walking	2.57 ± 2.58	6.33 ± 3.38	< 0.001	0.69 ± 1.11	3.61 ± 2.91	< 0.001
Walking up the stairs	2.03 ± 2.05	5.00 ± 2.52	< 0.001	0.76 ± 1.20	3.72 ± 3.10	< 0.001
Exercising	4.03 ± 4.40	8.06 ± 5.06	< 0.001	2.10 ± 3.62	5.44 ± 4.16	< 0.001
Total	18.43 ± 14.15	42.78 ± 22.17	< 0.001	7.68 ± 8.41	32.56 ± 22.09	< 0.001

**Table 8:** SF-36 scores (mean ± SD) for satisfied and dissatisfied participants

Domain	1 week			1 month		
	Satisfied	Dissatisfied	*p*	Satisfied	Dissatisfied	*p*
Physical functioning	58.25 ± 21.72	45.56 ± 11.87	0.006	75.97 ± 19.96	57.22 ± 18.41	< 0.001
Role-physical	42.82 ± 21.63	29.86 ± 16.12	0.017	59.23 ± 23.77	41.67 ± 20.11	0.001
Bodily pain	62.20 ± 21.58	40.74 ± 22.55	< 0.001	77.66 ± 17.21	56.17 ± 24.84	< 0.001
General health	57.86 ± 15.57	50,56 ± 11.10	0.048	59.32 ± 16.19	54.44 ± 12.11	**0.220**
Vitality	66.17 ± 16.05	53.47 ± 19.32	0.008	70.16 ± 15.12	59.38 ± 14.42	0.007
Social function	68.39 ± 23.01	45.14 ± 26.13	< 0.001	79.78 ± 18.74	59.72 ± 25.20	0.001
Role-emotional	57.36 ± 25.23	41.67 ± 27.56	0.025	67.58 ± 24.49	51.39 ± 25.12	0.005
Mental health	75.98 ± 16.33	65.83 ± 22.44	**0.068**	76.57 ± 16.31	70.83 ± 19.80	**0.311**
PCS	55.28 ± 15.61	41.68 ± 10.91	< 0.001	68.04 ± 15.38	52.38 ± 14.34	< 0.001
MCS	66.97 ± 15.96	51.53 ± 13.29	0.001	73.52 ± 15.44	60.33 ± 18.97	0.002

*MCS* mental component summary score, *PCS* physical component summary score

Note: significant differences between the means are marked in bold

The principal component analysis identified 3 components with a relatively good distribution of variance, with the first component accounting for 56% of the variance. Identical results were obtained in the separate inguinal hernia and other abdominal hernia groups. The loading weights for the first component ranged from 0.402 to 0.840, irrespective of the hernia group (Table 9).

**Table 9:** Component matrix (loading weights of 3 extracted components)

Domain	Content	Component		
		1	2	3
Sitting up	Pain	0.840	− 0.146	− 0.033
Bending over	Movement limitations	0.839	− 0.239	0.032
Activities of daily living	Pain	0.838	− 0.274	0.085
Bending over	Pain	0.826	− 0.183	0.013
Walking	Movement limitations	0.810	− 0.339	0.001
Walking up the stairs	Pain	0.806	− 0.263	0.028
Sitting up	Movement limitations	0.806	− 0.244	0.010
Walking up the stairs	Movement limitations	0.800	− 0.304	0.074
Activities of daily living	Movement limitations	0.796	− 0.336	0.053
Coughing or deep breathing	Movement limitations	0.795	− 0.296	0.041
Coughing or deep breathing	Pain	0.789	− 0.200	0.056
Walking	Sensation of mesh	0.786	0.387	− 0.220
Walking	Pain	0.783	− 0.331	− 0.013
Coughing or deep breathing	Sensation of mesh	0.778	0.397	− 0.196
Walking up the stairs	Sensation of mesh	0.774	0.367	− 0.219
Sitting up	Sensation of mesh	0.768	0.407	− 0.299
Activities of daily living	Sensation of mesh	0.739	0.487	− 0.254
Bending over	Sensation of mesh	0.736	0.495	− 0.157
Laying down	Pain	0.645	− 0.137	− 0.151
Laying down	Sensation of mesh	0.631	0.364	− 0.354
Exercising	Movement limitations	0.514	0.211	0.806
Exercising	Pain	0.486	0.256	0.801
Exercising	Sensation of mesh	0.402	0.604	0.658

## Discussion

In this study, we have presented results for the translation, cross-cultural adaption, and validation of the Lithuanian version of the CCS. Our findings add to those of other investigators who have shown that CCS is a valid and specific instrument for measuring QoL after hernia surgery using mesh [1, 3, 4, 20].

Following the publication of the initial validation study in 2007, CCS has been translated into 28 languages and is currently being used in more than 45 countries throughout the world. In some countries, CCS has been included in national and international registries [21–23].

This study confirms the relevance and importance of QoL investigations after hernia repair and their value for comparing patient outcomes at the personal, institutional, and international levels. However, despite the high number of language translations, only the Dutch have carried out an adaptation study [1]. A possible reason for this is that studies conducted in the language of a small population are only published in local journals because they are of less interest to international journals. In our experience, however, the translation and validation of questionnaires according to recommended guidelines are important steps for their widespread usage. International standardization of findings is important for the communication and comparison of cross-cultural results. The Lithuanian version of CCS will allow Lithuanian surgeons to participate in international hernia clinical trials.

As expected, the results of this study suggest that CCS has robust internal consistency for the total scale, independent of the hernia type. Deletion of individual items in the questionnaire responses did not increase the Cronbach’s α value, indicating that all questions were equally reliable and that none adversely affect the overall reliability of the questionnaire.

Test–retest reliability showed strong and significant correlations between the two assessments. Correlations for several items were very close to reaching a level of > 0.4. These findings are similar to those of Nielsen et al. [1]. Recovery after hernia surgery is a dynamic process and hence the condition of participants can improve significantly during the first 3 weeks postoperatively and prior to the second assessment. However, if a shorter period is chosen for the second assessment then there is a high probability that participants will remember the previous answers. Heniford et al. [4] found a stronger test–retest correlation compared to our results. This may be explained by the fact that questionnaires in their study could be submitted at a later stage in the recovery period where changes in the condition of participants is much slower. For the same reason, the loading weight provided by the last activity (while exercising) in the first component differs from that of the original version. In contrast to the findings of Heniford et al., we found the highest weight was provided by the activities of sitting up, bending over and walking. The lowest weight was provided by the last activity (exercising), since participants avoided exercise in the early postoperative period. However, even the smallest weights in our study were > 0.4 (Table 9). In the study by Heniford et al. [3], vice versa exercise-related questions had the highest weights. This supports the deduction that weight could be distributed differently at various time points and hence all 23 questions in the survey are not redundant.

It has already been shown that disease-specific questionnaires are superior in identifying problems and more sensitive at detecting changes caused by disease-specific conditions, especially after surgical treatment [3, 5, 6]. The observed correlations between total CCS score and different domains of SF-36 confirms the findings of Nielsen et al. [1] that CCS only weakly reflects the general health and mental health components. However, strong correlations were found with the domains of bodily pain, physical functioning, and consequently with the physical component summary score. In the discriminant validity assessment, we found clear differences between satisfied and unsatisfied participants for all domains of CCS, whereas no differences in mental health and general health domains were found at 1 month with the SF-36 questionnaire. In summary, our findings support the view that hernia surgery has a limited impact on mental health, role functioning and social functioning and that SF-36 is less suitable to assess outcomes in patients after hernia surgery [24–26].

This study has several limitations. The inguinal hernia patients comprised the majority of cases and were mostly male. Moreover, analyzing 36 cases we did not reach the recommended sample size in the “other hernias” group [27]. Nevertheless, the most correlations and differences were statistically significant as in the other validation study of original and Dutch CCS, which included only 15 cases of not inguinal hernias and showed similar results [1]. Despite the statistical significance of the data, the conclusion that CCS is reliable and valid for “other hernia” group must be interpreted with caution. Further data is needed to clarify it.

In conclusion, the adapted and validated Lithuanian CCS presented in this study is a short, feasible, and effective questionnaire for measuring QoL following surgical repair of inguinal hernias with mesh.

We suggest the use of CCS as a standardized tool for the evaluation and comparison of QoL following inguinal hernia surgery with mesh in personal, local and international contexts.

## Supplementary Information

Below is the link to the electronic supplementary material.

Supplementary file1 (DOCX 16 kb)
