# Supplementary material for: Adaptation and validation of the Carolinas Comfort Scale: a questionnaire-based cross-sectional study
Source: Hernia. 2021 Mar 29;26(3):735–44. doi: 10.1007/s10029-021-02399-4 (PMC9200669; doi:10.1007/s10029-021-02399-4)
Supplement: Supplementary file 1 — Supplementary file1 (DOCX 16 kb) [file 10029_2021_2399_MOESM1_ESM.docx]

**Appendix 1** Carolinas Comfort Scale (English and Lithuanian versions)

| English | Lithuanian |
| --- | --- |
| Please answer ALL questions for each of the 8 activities. | Atsakykite į VISUS klausimus apie kiekvieną iš 8 veiklų. |
| Use N/A if an activity was not performed. | Jei veikla nebuvo atliekama, naudokite N/A. |

|  | English | Lithuanian |
| --- | --- | --- |
| 0= | No Symptoms | Nėra simptomų |
| 1= | Mild but not bothersome symptoms | Lengvi, bet neįkyrūs simptomai |
| 2= | Mild and bothersome symptoms | Lengvi ir įkyrūs simptomai |
| 3= | Moderate and/or daily symptoms | Vidutinio sunkumo ir (arba) kasdieniai simptomai |
| 4= | Severe symptoms | Sunkūs simptomai |
| 5= | Disabling symptoms | Negalią sukeliantys simptomai |

|  | English | Lithuanian |  | | | | | | |
| --- | --- | --- | --- | --- | --- | --- | --- | --- | --- |
| **1.** | **While laying down, do you have** | **Ar gulėdami Jūs** |  | | | | | | |
| a) | sensation of mesh | jaučiate tinklelį | 0 | 1 | 2 | 3 | 4 | 5 | N/A |
| b) | pain | patiriate skausmą | 0 | 1 | 2 | 3 | 4 | 5 | N/A |
| **2.** | **While bending over, do you have** | **Ar pasilenkdami Jūs** |  | | | | | | |
| a) | sensation of mesh | jaučiate tinklelį | 0 | 1 | 2 | 3 | 4 | 5 | N/A |
| b) | pain | patiriate skausmą | 0 | 1 | 2 | 3 | 4 | 5 | N/A |
| c) | movement limitations | patiriate judesių apribojimą | 0 | 1 | 2 | 3 | 4 | 5 | N/A |
| **3.** | **While sitting up, do you have** | **Ar sėdėdami Jūs** |  | | | | | | |
| a) | sensation of mesh | jaučiate tinklelį | 0 | 1 | 2 | 3 | 4 | 5 | N/A |
| b) | pain | patiriate skausmą | 0 | 1 | 2 | 3 | 4 | 5 | N/A |
| c) | movement limitations | patiriate judesių apribojimą | 0 | 1 | 2 | 3 | 4 | 5 | N/A |
| **4.** | **While performing activities of daily living (i.e. getting out of bed, bathing, getting dressed), do you have** | **Ar užsiimdami kasdiene veikla (t. y., kai keliatės iš lovos, maudotės, rengiatės) Jūs** |  | | | | | | |
| a) | sensation of mesh | jaučiate tinklelį | 0 | 1 | 2 | 3 | 4 | 5 | N/A |
| b) | pain | patiriate skausmą | 0 | 1 | 2 | 3 | 4 | 5 | N/A |
| c) | movement limitations | patiriate judesių apribojimą | 0 | 1 | 2 | 3 | 4 | 5 | N/A |
| **5.** | **When coughing or deep breathing, do you have** | **Ar kosėdami arba giliai kvėpuodami Jūs** |  | | | | | | |
| a) | sensation of mesh | jaučiate tinklelį | 0 | 1 | 2 | 3 | 4 | 5 | N/A |
| b) | pain | patiriate skausmą | 0 | 1 | 2 | 3 | 4 | 5 | N/A |
| c) | movement limitations | patiriate judesių apribojimą | 0 | 1 | 2 | 3 | 4 | 5 | N/A |
| **6.** | **While walking, do you have** | **Ar vaikščiodami Jūs** |  | | | | | | |
| a) | sensation of mesh | jaučiate tinklelį | 0 | 1 | 2 | 3 | 4 | 5 | N/A |
| b) | pain | patiriate skausmą | 0 | 1 | 2 | 3 | 4 | 5 | N/A |
| c) | movement limitations | patiriate judesių apribojimą | 0 | 1 | 2 | 3 | 4 | 5 | N/A |
| **7.** | **When walking up the stairs, do you have** | **Ar lipdami laiptais aukštyn Jūs** |  | | | | | | |
| a) | sensation of mesh | jaučiate tinklelį | 0 | 1 | 2 | 3 | 4 | 5 | N/A |
| b) | pain | patiriate skausmą | 0 | 1 | 2 | 3 | 4 | 5 | N/A |
| c) | movement limitations | patiriate judesių apribojimą | 0 | 1 | 2 | 3 | 4 | 5 | N/A |
| **8.** | **While exercising, do you have** | **Ar mankštindamiesi Jūs** |  | | | | | | |
| a) | sensation of mesh | jaučiate tinklelį | 0 | 1 | 2 | 3 | 4 | 5 | N/A |
| b) | pain | patiriate skausmą | 0 | 1 | 2 | 3 | 4 | 5 | N/A |
| c) | movement limitations | patiriate judesių apribojimą | 0 | 1 | 2 | 3 | 4 | 5 | N/A |
